# Supplementary material for: Case-control study on determinants of uterine rupture among mothers who gave birth at Hawassa University comprehensive specialized hospital
Source: PLoS One. 2023 Jan 13;18(1):e0279175. doi: 10.1371/journal.pone.0279175 (PMC9838871; doi:10.1371/journal.pone.0279175)
Supplement: Attachment — Submitted filename: CASP-Case-Control-Study-Checklist-2018-fillable-form.pdf [file pone.0279175.s003.pdf]

CASP
Critical Appraisal Skills Programme

www.casp-uk.net
info@casp-uk.net
Summertown Pavilion, Middle Way Oxford OX2 7LG

CASP Checklist: 11 questions to help you make sense of a **Case Control Study**

How to use this appraisal tool: Three broad issues need to be considered when appraising a case control study:

- Are the results of the study valid? (Section A)
- What are the results? (Section B)
- Will the results help locally? (Section C)

The 11 questions on the following pages are designed to help you think about these issues systematically. The first three questions are screening questions and can be answered quickly. If the answer to both is "yes", it is worth proceeding with the remaining questions. There is some degree of overlap between the questions, you are asked to record a "yes", "no" or "can't tell" to most of the questions. A number of italicised prompts are given after each question. These are designed to remind you why the question is important. Record your reasons for your answers in the spaces provided.

About: These checklists were designed to be used as educational pedagogic tools, as part of a workshop setting, therefore we do not suggest a scoring system. The core CASP checklists (randomised controlled trial & systematic review) were based on JAMA 'Users' guides to the medical literature 1994 (adapted from Guyatt GH, Sackett DL, and Cook DJ), and piloted with health care practitioners.

For each new checklist, a group of experts were assembled to develop and pilot the checklist and the workshop format with which it would be used. Over the years overall adjustments have been made to the format, but a recent survey of checklist users reiterated that the basic format continues to be useful and appropriate.

Referencing: we recommend using the Harvard style citation, i.e.: *Critical Appraisal Skills Programme (2018). CASP (insert name of checklist i.e. Case Control Study) Checklist. [online] Available at: URL. Accessed: Date Accessed.*

©CASP this work is licensed under the Creative Commons Attribution – Non-Commercial-Share A like. To view a copy of this license, visit http://creativecommons.org/licenses/by-nc-sa/3.0/ www.casp-uk.net

Critical Appraisal Skills Programme

Paper for appraisal and reference:

Section A: Are the results of the trial valid?

1. Did the study address a clearly focused issue?

Yes ✔
Can't Tell ☐
No ☐

HINT: An issue can be 'focused' In terms of
- the population studied
- Whether the study tried to detect a beneficial or harmful effect
- the risk factors studied

Comments: The paper did not describe how they selected 194 cases from 247 total uterine rupture

2. Did the authors use an appropriate method to answer their question?

Yes ✔
Can't Tell ☐
No ☐

HINT: Consider
- Is a case control study an appropriate way of answering the question under the circumstances
- Did it address the study question

Comments: However, prevalence of uterine rupture can not be answered through hospital based study

Critical Appraisal Skills Programme

Is it worth continuing?

3. Were the cases recruited in an acceptable way?

Yes ☐
Can't Tell ☑
No ☐

HINT: We are looking for selection bias which might compromise validity of the findings

- are the cases defined precisely
- were the cases representative of a defined population (geographically and/or temporally)
- was there an established reliable system for selecting all the cases
- are they incident or prevalent
- is there something special about the cases
- is the time frame of the study relevant to disease/exposure
- was there a sufficient number of cases selected
- was there a power calculation

Comments:

4. Were the controls selected in an acceptable way?

Yes ☑
Can't Tell ☐
No ☐

HINT: We are looking for selection bias which might compromise the generalisability of the findings

- were the controls representative of the defined population (geographically and/or temporally)
- was there something special about the controls
- was the non-response high, could non-respondents be different in any way
- are they matched, population based or randomly selected
- was there a sufficient number of controls selected

Comments:

Critical Appraisal Skills Programme

5. Was the exposure accurately measured to minimise bias?

Yes ☐
Can't Tell ☑
No ☐

HINT: We are looking for measurement, recall or classification bias

- was the exposure clearly defined and accurately measured
- did the authors use subjective or objective measurements
- do the measures truly reflect what they are supposed to measure (have they been validated)
- were the measurement methods similar in the cases and controls
- did the study incorporate blinding where feasible
- is the temporal relation correct (does the exposure of interest precede the outcome)

Comments:

6. (a) Aside from the experimental intervention, were the groups treated equally?

HINT: List the ones you think might be important, that the author may have missed

- genetic
- environmental
- socio-economic

List:

6. (b) Have the authors taken account of the potential confounding factors in the design and/or in their analysis?

Yes ☐
Can't Tell ☐
No ☑

HINT: Look for

- restriction in design, and techniques e.g. modelling, stratified-, regression-, or sensitivity analysis to correct, control or adjust for confounding factors

Comments: please perform sensitivity analysis

Section B: What are the results?

7. How large was the treatment effect?

HINT: Consider
- what are the bottom line results
- is the analysis appropriate to the design
- how strong is the association between exposure and outcome (look at the odds ratio)
- are the results adjusted for confounding, and might confounding still explain the association
- has adjustment made a big difference to the OR

Comments:

the effect was not precise and the interval was very hide, suggesting unidentified confounders

8. How precise was the estimate of the treatment effect?

HINT: Consider
- size of the p-value
- size of the confidence intervals
- have the authors considered all the important variables
- how was the effect of subjects refusing to participate evaluated

Comments:

CASP
Critical Appraisal
Skills Programme

9. Do you believe the results?

Yes ✔
No ☐

HINT: Consider
- big effect is hard to ignore!
- Can it be due to chance, bias, or confounding
- are the design and methods of this study sufficiently flawed to make the results unreliable
- consider Bradford Hills criteria (e.g. time sequence, does-response gradient, strength, biological plausibility)

Comments: high likely of bias in the study

Section C: Will the results help locally?

10. Can the results be applied to the local population?

Yes ☐
Can't Tell ☐
No ✔

HINT: Consider whether
- the subjects covered in the study could be sufficiently different from your population to cause concern
- your local setting is likely to differ much from that of the study
- can you quantify the local benefits and harms

Comments:

11. Do the results of this study fit with other available evidence?

Yes ✔
Can't Tell ☐
No ☐

HINT: Consider
- all the available evidence from RCT's Systematic Reviews, Cohort Studies, and Case Control Studies as well, for consistency

Comments: the listed factors were listed in previous sources

Remember One observational study rarely provides sufficiently robust evidence to recommend changes to clinical practice or within health policy decision making. However, for certain questions observational studies provide the only evidence. Recommendations from observational studies are always stronger when supported by other evidence.
