# Supplementary material for: Case-control study on determinants of uterine rupture among mothers who gave birth at Hawassa University comprehensive specialized hospital
Source: PLoS One. 2023 Jan 13;18(1):e0279175. doi: 10.1371/journal.pone.0279175 (PMC9838871; doi:10.1371/journal.pone.0279175)
Supplement: Attachment — Submitted filename: [reviewers report] Case-control study on determinants of uterine rupture among mothers who gave birth.docx [file pone.0279175.s002.docx]

**Case-control study on determinants of uterine rupture among mothers who gave birth at Hawassa university comprehensive specialized hospital.**

**SUMMARY**

**Overview of the strengths and weaknesses**

| Strength | Location | Weakness | page | Recommendation |
| --- | --- | --- | --- | --- |
| Precise; containing what, who, where | **Title** |  |  |  |
| Contains the study design |  |  |  |  |
|  | **Abstract** |  |  |  |
| Provided a balanced summary of what was done and found |  | Confidence intervals not included with the odd ratios | ii | Add the 95%CI to the odd ratios |
|  | **Introduction** |  |  |  |
| Background/rationale looks scientific and rationale for investigation reported. |  | Objective looks like a justification. | 2 | If this is the objective, it may need to be re-written... like "This study assessed.......". If not, it might need to be stated as suggested. |
|  | **Methods** |  |  |  |
|  |  | ***Sampling participants*** |  |  |
| Study design was clearly stated and the study setting, locations, duration of data collection were reported. |  | Sampling frame may not be necessary if probability sampling method is not used. | 2 | If a probabilistic sampling is used, it should be clearly stated and adequately described. |
| Study participants were clearly reported. |  | **Data collection tools and procedure** |  |  |
| Sample size was calculated |  | “…tasted” seems to be a typographical error | 3 | May be changed to “tested” or better still, “pre-tested”. |
| Inclusion and exclusion criteria stated. |  |  |  |  |
| Data management and analysis are quite explicit |  |  |  |  |
|  | **Results** |  |  |  |
| Descriptive data well-presented generally |  | **Trends of uterine rupture** |  |  |
| Presented the unadjusted odds ratio and the criteria for adjustment and inclusion in the multivariable regression stated |  | The prevalence estimate of 1.09% description not sufficient. | 3 | Adding 95%CI to this prevalence may be better? |
|  |  | Test statistics not reported in comparing the differences between the cases and controls in Tables 1,2,3and 4 | 4,5,6,7,8 | Since it is a case-control study, there may be the need to report if there is a significant difference between the two groups. if cross-tabs, you may need to state the chi-square estimates, crude odds and p-values. |
|  |  | Total number eventually studied for each variable are not reported to determine missing data. | 4,5,6,7,8 | The total number studied for cases and control might be necessary to be added either at the top if all or almost all share similar total numbers or added individually at the level of each variable to detect missing data. |
|  |  | Range may not be necessary to be reported.  (Table 1 -under age) | 4 | Since the observation appears normally distributed, mean(SD) should be sufficient without the range. I will suggest that the range value be removed. |
|  |  | Meaning of TOLAC may not be clear to a lay-reader (Table 2) | 6 | It may be necessary to state the full meaning at the base of the table. |
|  |  | **Health system-related characteristics** |  |  |
|  |  | Since this observation appears not normally distributed, mean (SD) should not be reported. | 7 | I will suggest that Median (interquartile range) be reported. |
|  |  | Since this observation appears not normally distributed, mean (SD) should not be reported. (Table 3 under “duration between  admission and surgery” and “Duration of hospital  stay” | 7 | I will suggest that Median(interquartile range) be reported. |
|  |  | Range may not be necessary since the observation appears normally distributed.  (Table 4- under “weight if the baby” | 8 | May be removed. |
|  |  | The meaning of “LUST”, “TAH” and “STH” may not be clear to a lay-reader. (Table 4) | 8 | The full-meaning should be provided at the base of the table. |
|  |  | **Determinants of uterine rupture** |  |  |
|  |  | “Having ANC follow up” seems not intended. |  | It looks like you meant "Having no ANC follow-up"? check this up by comparing with your table and make necessary correction. |
|  |  | The reported AOR and their 95%CI seems not corresponding with the results shown on the Table 5 for “Number of ANC follow up (less than four ANC)”; “gravidity more than 5” and “fetal weight greater than 4000 gram” | 9 | Please, harmonize these results. |
|  |  | For “other medical or  obstetric disorders” crude OR was reported as against the AOR. | 9 | Please, report the adjusted OR instead |
|  |  |  |  |  |
|  | **Discussion** |  |  |  |
| Key results presented and compared with other similar studies. Also stated the implication of the similarities and/or public health implications of each of the result. |  | Paragraph 1 and 2, though discussing the same point are separate. | 10 | the two paragraphs can be merged since they are focused on a particular result |
| Limitation of the study clearly stated. |  | Paragraph 3 and 4, though discussing the same point are separate. | 10 | the two paragraphs can be merged since they are focused on a particular result |
| It gives a cautious view of the generalizability of the study result. |  |  |  |  |
|  |  | **References** |  |  |
|  |  | Reference 8 appears not properly done | 15 |  |

**Overall Recommendation**

Uterine rupture is one of the significant causes of postpartum haemorrhage and identifying its associated factors is a step towards preventing and controlling maternal mortality especially in low- and middle income countries.

The manuscript is well-thought out and addresses an issue of public health importance. Its scientific and analytic approach is sound. It will just require minor analytic and editing revisions to improve its quality.
